# Understanding the Interplay of Dietary Intake and Eating Behavior in Type 2 Diabetes

**DOI:** 10.3390/nu16060771

**Published:** 2024-03-08

**Authors:** Ana Maria Gal, Camelia Oana Iatcu, Alina Delia Popa, Lidia Iuliana Arhire, Laura Mihalache, Andreea Gherasim, Otilia Nita, Raluca Meda Soimaru, Roxana Gheorghita, Mariana Graur, Mihai Covasa

**Affiliations:** 1 Faculty of Medicine, “Grigore T. Popa” University of Medicine and Pharmacy, 700115 Iasi, Romania; ana-maria.a.ilisei@d.umfiasi.ro (A.M.G.); oana.iatcu@usm.ro (C.O.I.); alina.popa@umfiasi.ro (A.D.P.); laura.mihalache@umfiasi.ro (L.M.); andreea.gherasim@umfiasi.ro (A.G.); otilia.nita@umfiasi.ro (O.N.); raluca-meda-m-frigura@d.umfiasi.ro (R.M.S.); 2 Department of Biomedical Sciences, Faculty of Medicine and Biological Sciences, Stefan cel Mare University, 720229 Suceava, Romania; roxana.puscaselu@usm.ro (R.G.); mariana.graur@usm.ro (M.G.); mcovasa@westernu.edu (M.C.)

**Keywords:** diabetes mellitus, T2DM, EPIC FFQ, DEBQ, emotional eating, external eating, restrained eating

## Abstract

Diet and eating behavior both play a crucial role in the prevention and management of type 2 diabetes mellitus (T2DM). The main objective of this study was to investigate the relationship between dietary intake and eating behavior in a population of patients with T2DM. A cross-sectional study was performed using 416 patients with T2DM and their dietary intake and eating behavior were assessed with validated questionnaires. Women scored significantly higher than men for emotional and restrained eating (*p* < 0.001). Correlation analyses showed that emotional eaters consumed significantly more calories (r = 0.120, *p* = 0.014) and fat (r = 0.101, *p* = 0.039), as well as non-alcoholic beverages for women (r = 0.193, *p* = 0.003) and alcohol for men (r = 0.154, *p* = 0.038). Also, individuals who ate based on external cues consumed significantly more calories (r = 0.188, *p* < 0.001) and fat (r = 0.139, *p* = 0.005). These results demonstrate that eating behavior influences dietary intake. Understanding this relationship could optimize diabetes management and allow for more individualized nutritional guidance.

## 1. Introduction

Type 2 diabetes mellitus (T2DM) is a chronic and complicated metabolic condition that is influenced by an individual’s age, lifestyle, diet, genetics, and environment [1]. Diabetes mellitus is one of the world’s most rapidly expanding diseases [2] and its high incidence places a significant burden on global public health systems [1]. Although significant technological and pharmaceutical advances have been made in diabetes management, diabetes control remains a challenge [3]. The significant impact of T2DM, which accounts for approximately 90% of the estimated 537 million cases of diabetes worldwide, is recognized on a global scale [4,5]. Changes in lifestyle, such as increased physical activity, nutritional approaches, smoking cessation, and maintaining a healthy body weight, are typically included in diabetes management strategies in order to reduce the negative effects of the disease [4]. With its impact on metabolism and body weight, diet plays a key role in the management and prevention of T2DM and diet therapy is regarded as the main strategy for managing diabetes [6]. However, medical practitioners and their patients are often uncertain about what nutritional recommendations are appropriate or what types of nutrition plans to use [7]. Long-term weight loss maintenance and adherence to restrictive eating habits represent a challenge for many patients. This can result in disappointment and adverse psychological and emotional consequences [7].

Eating behavior is a complicated process which involves interactions between physiological variables and the food environment [8]. Eating behavior refers not only to the foods consumed, the nutrients consumed, or the process of eating, but also to food preferences, choices, and consumption patterns [9]. Eating behavior may encompass different construct areas such as homeostatic eating (i.e., intuitive eating), reflective eating (i.e., mindful eating, dietary restrain), reactive eating (i.e., food craving, emotional eating, external eating), or disordered eating (i.e., binge eating disorders) [10].

T2DM and eating behavior are closely related [11] and different eating behaviors, such as restrained, emotional, and external eating, can impact the development and management of the disease [12,13,14]. Restrained eating in T2DM is often associated with rigid dietary control and may affect dietary intake [9]. Emotional eating, meaning eating in response to emotions rather than physiological signals such as hunger, and also external eating, influenced by external food cues, can lead to overeating and may also contribute to excessive and unhealthy food choices [13,15,16]. Emotional, restrained, and external eating behaviors have been associated with a high frequency of binge eating, which in turn affects weight management [17]. Moreover, most individuals are fearful of the thought of going on a “diet” in order to manage a chronic, progressive disease such as diabetes. However, this approach is often used since maintaining a healthy eating pattern and knowing what to eat can be challenging for most individuals [18]. The concept of a diabetic-friendly diet changes as nutritional science does. Thus, a patient with T2DM faces several barriers when following their diet. This may cause confusion and loss of interest and motivation to follow dietary recommendations [19]. In addition, some dietary restrictions are required not only to prevent T2DM, but also to improve metabolic control of the disease [20]. Dietary restrictions can have positive effects, such as improved glycemic control and weight loss [21], improved cardiovascular wellness [22], and improved quality of life [23], but they can also have negative effects. Among these are the potential nutritional deficits, increased risk of hypoglycemia, and various psychological effects that impact eating behavior, such as feelings of guilt, shame, or social isolation [24]. Both emotional and behavioral manifestations influence how individuals eat [25].

In addition to analyzing food consumption, it is critical to evaluate other factors that influence changes in dietary intake and to design dietary interventions tailored to the needs of different populations, both to maintain optimal health and to support the care of individuals affected by the disease [26]. Understanding the dietary behavior of patients with T2DM, in addition to the factors that affect their food choices, would make nutrition education and recommendations more effective. This could also help close the gap between what patients with T2DM know about what to eat and what they actually eat in their everyday lives [9]. To examine this, our study was designed to investigate the relationship between eating behavior (emotional, restrained, and external) and dietary intake in a population of patients with T2DM.

## 2. Materials and Methods

### 2.1. Study Design

Patients with diagnosed T2DM from the Clinical Centre for Diabetes, Nutrition and Metabolic Diseases of St. Spiridon’s Hospital, Iasi, Romania participated in a cross-sectional study. The selection of participants was non-random, and all patients who presented for their regular follow-ups with the diabetes specialist were informed about the study and were invited to participate. The criteria for inclusion in the study were: adults over the age of 18 years, diagnosed with T2DM, and with or without specific associated medication. Patients without T2DM, who were pregnant or nursing, who were treated with insulin, who had documented cognitive problems, hearing disorders, eating behavior disorders, or other pathologies that could have influenced dietary intake (i.e., cancer, chronic kidney disease), who declared following a special diet, or who had incomplete questionnaire responses were all excluded.

### 2.2. Data Collection

#### 2.2.1. Measurement of Demographic and Anthropometric Data

Age, date of birth, place of living (urban or rural), occupation, and smoking status were declared by the participants before completing the questionnaires.

The following anthropometric data were measured: weight and height. Weight was measured to the nearest 0.1 kg with a digital portable scale, without shoes and with light clothing; height was measured with a stadiometer, without shoes. Body Mass Index (BMI) was calculated according to the formula weight/height^2^ (kg/m^2^). Based on the BMI classification of the World Health Organization [27], people were divided into: underweight (BMI < 18.5 kg/m^2^), normal weight (BMI between 18.5–24.9 kg/m^2^), overweight (BMI between 25.0–29.9 kg/m^2^), with obesity (BMI > 30.0 kg/m^2^).

#### 2.2.2. Measurement of Dietary Intake

The EPIC food frequency questionnaire (EPIC-FFQ) [28] was administered by a trained dietitian to assess the dietary intake of participants. The questionnaire is divided into two sections. Section 1 is a 130-item food list (individual foods, combinations of individual foods, or food types) with a portion size attached: medium serving, standard unit, or household measure. Participants in the study were asked to choose an appropriate frequency of consumption for each item from the nine frequency categories, recalling the 12 months prior to participation. The data were manually entered into a spreadsheet using numeric codes ranging from “1” for “never or less than once a month” to “9” for “6+ times each day”. A “−9” code was used to indicate data instances where no frequency data were collected. The second section of the questionnaire included specific questions about cereal, milk, and fat consumption.

Average daily intakes were calculated based on the consumption frequency and portion size with the FFQ Epic Tool For Analysis (FETA), version 6 [28], a tool that translates dietary information from the FFQ into energy and nutrients intake. The FETA program generated a report that included 46 nutrients and 14 food groups, of which the following were retained for further analysis: daily energy intake expressed in kcalories (Kcal) and daily intake of macronutrients expressed in grams (g) or milligrams (mg): carbohydrates (g), fiber (g), proteins (g), total lipids (g), saturated fatty acids (SFA) (g), monounsaturated fatty acids (MUFA) (g), polyunsaturated fatty acids (PUFA) (g), cholesterol (mg), and alcohol (g). Based on these, the percentages for each macronutrient relative to the daily energy intake were calculated. The food groups obtained by analyzing data from the EPIC-FFQ questionnaire were: “Alcoholic beverages”, “Cereals and cereal products”, “Eggs and eggs dishes”, “Fats and oils”, “Fish & fish products”, “Fruit”, “Meat and meat products”, “Milk and milk products”, “Non-alcoholic beverages”, “Nuts and seeds”, “Potatoes”, “Soups & sauces”, “Sugars; preserves and snacks”, and “Vegetables”. The EPIC-FFQ was previously translated and validated into Romanian [29].

#### 2.2.3. Measurement of Eating Behavior

The Dutch Eating Behavior Questionnaire (DEBQ), developed by Van Strein T. in 1986 [30], was applied by a trained interviewer to evaluate the patients’ eating behavior. The questionnaire has 33 items and five scales: three main scales: “Emotional eating” (EmoE) with 13 items (overeating due to emotions), “External eating” (ExtE) with 10 items (eating in response to food-related stimuli regardless of hunger and satiety), and “Restrained eating” (RE) with 10 items (attempts to refrain from eating); “Diffuse emotions” (DEmo) and “Clearly labelled emotions” (CLEmo) are subscales for the “Emotional eating” scale. All items are scored on a five-point Likert scale from 1 (“never”) to 5 (“very often”). A raw score was calculated for each scale and subscale, and higher scores on each subscale and scale reflect a higher level of emotional, external, or restrained eating. The DEBQ was previously translated and validated in Romanian for the general population [31].

### 2.3. Ethical Consideration

This study was conducted in accordance with the ethical standards of the Declaration of Helsinki and was approved by the Ethics Commission of Gr. T. Popa University (82/20.05.2021). All participants who agreed to participate in the study signed informed consent. The study did not involve any risk and did not exert any physical or mental discomfort on the included participants. Participants were not remunerated or compensated in any way for their participation in the study.

### 2.4. Statistical Analysis

The data collected were numerically encoded and centralized in a Microsoft Excel 2010 database. For statistical analysis, version 20 of the SPSS (Statistical Package for the Social Sciences) program was used. Continuous variables were expressed as mean ± standard deviation (SD), and categorical variables as numbers and proportions. Chi-squared and *t*-tests were used to compare sample characteristics by sex. The Levene test was used to check the homogeneity of variances. The normality of the variable distribution was verified using the Kolmogorov–Smirnov test. To compare mean differences between groups, One-Way ANOVA was used, and to evaluate relationships between dietary intake and the five scales measured for eating behavior, Pearson correlation coefficients (r) were computed. A *p* value less than 0.05 was considered statistically significant.

## 3. Results

### 3.1. General and Anthropometric Characteristics of the Studied Population

The study population included 416 T2DM patients, of which 56.7% (*n* = 236) were women. The average weight of the men was significantly higher than the average weight of women (95.08 ± 17.64 vs. 81.12 ± 14.98, *p* < 0.001) in this study, but no significant differences were observed between the men and women for age or BMI. The general characteristics of the study population are presented in Table 1.

### 3.2. Dietary Intake in the Studied Population

The mean energy intake was 1584.78 ± 605.57 kcal, with the men having a significantly higher energy intake than the women (*p* = 0.003). In the studied population, the men also recorded a significantly higher intake of total lipids (*p* = 0.044), saturated fatty acids (*p* = 0.02), cholesterol (*p* < 0.001), and proteins (*p* < 0.001). The means for the percentages of macronutrients consumed of the total energy intake were: 50.05% ± 8.33 for carbohydrate intake, 20.77% ± 3.55 for protein intake, and 31.74% ± 6.81 for total lipid intake. Significant differences (*p* = 0.004) between men and women were observed only for the percentage intake of carbohydrates, with the women having a significantly higher intake than men (51.09% vs. 48.70%, *p* < 0.05). Regarding food groups, the men had a significantly higher intake of cereals and cereal products (*p* = 0.013), potatoes (*p* = 0.045), meat and meat products (*p* = 0.004), fish and fish products (*p* = 0.004), eggs and eggs dishes (*p* < 0.001), soups and sauces (*p* = 0.006), and alcoholic beverages (*p* < 0.001), while the women had a higher consumption of fruits (*p* = 0.016), nuts and seeds (*p* = 0.020), and non-alcoholic beverages (*p* < 0.001) (Table 2).

### 3.3. Eating Behavior in the Studied Population

Women scored significantly higher than the men in all scales (*p* < 0.001), except for the ExtE scale (*p* > 0.05). Depending on the BMI status, statistically significant differences were observed only for RE (*p* < 0.001), with patients with obesity and who were overweight having higher scores compared to those with normal weight. Higher scores were also noted for individuals living in an urban area for the scales corresponding to emotional eating (DEmo: *p* = 0.017; CLEmo: *p* = 0.014; EmoE: *p* = 0.012) and restrained eating (*p* = 0.045) (Table 3).

### 3.4. Correlations between Food Intake and Eating Behavior in the Studied Group

**Figure 1:**
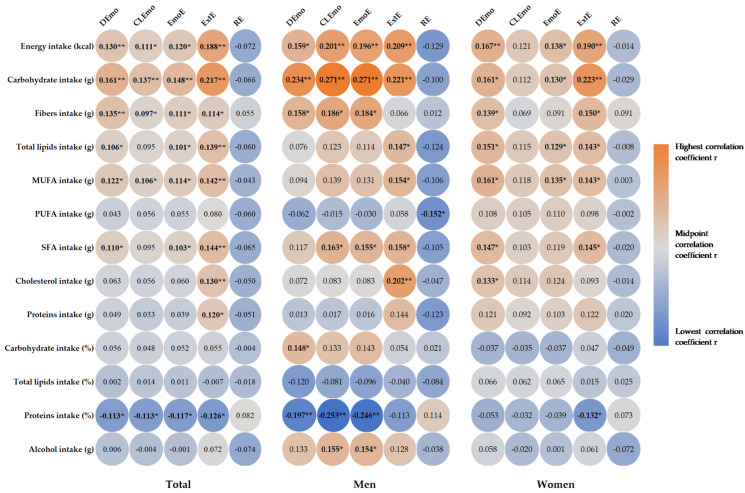
Heat map of correlation between energy and macronutrients intake and eating behavior. Correlation coefficient r is presented in circle, and the strength of the correlation is depicted in color, with orange being the highest and blue the lowest correlation coefficient: * *p* < 0.05, ** *p* < 0.01. DEmo = diffuse emotions, CLEmo = clearly labelled emotions, EmoE = emotional eating, ExtE = external eating, RE = restrained Eating, MUFA = monounsaturated fatty acids, PUFA = polyunsaturated fatty acids, SFA = saturated fatty acids.

Correlations between participants’ daily dietary intake of energy and macronutrients are presented in Figure 1. We found a positive correlation between emotional eating and external eating and dietary intake. Emotional eaters and external eaters showed a significantly higher intake of energy (DEmo: r = 0.130, *p* = 0.008; CLEmo: r = 0.111, *p* = 0.024; EmoE: r = 0.120, *p* = 0.014; ExtE: r = 0.188, *p* < 0.001), carbohydrates (DEmo: r = 0.161, *p* = 0.001; CLEmo: r = 0.137, *p* = 0.005; EmoE: r = 0.148, *p* = 0.002; ExtE: r = 0.217, *p* < 0.001), fiber (DEmo: r = 0.135, *p* = 0.006; CLEmo: r = 0.097, *p* = 0.048; EmoE: r = 0.111, *p* = 0.023; ExtE: r = 0.114, *p* = 0.020), total lipids (DEmo: r = 0.106, *p* = 0.031; EmoE: r = 0.101, *p* = 0.039; ExtE: r = 0.139, *p* = 0.005), SFA (DEmo: r = 0.110, *p* = 0.025; EmoE: r = 0.103, *p* = 0.036; ExtE: r = 0.144, *p* = 0.003), and MUFA (DEmo: r = 0.122, *p* = 0.013; CLEmo: r = 0.106, *p* = 0.031; EmoE: r = 0.114, *p* = 0.020; ExtE: r = 0.142, *p* = 0.004). In addition, external eaters showed a significantly higher intake of cholesterol (r = 0.130, *p* = 0.008) and proteins (r = 0.120, *p* = 0.014). Emotional eaters and external eaters had a significantly lower intake of proteins (in percentage) (DEmo r = −0.113, *p* = 0.021; CLEmo r = −0.113, *p* = 0.021; EmoE r = −0.117, *p* = 0.017; ExtE r = −0.126, *p* = 0.010), with no significant correlations for restrained eaters (*p* > 0.05).

In the men, emotional eaters showed a significantly higher intake of energy (DEmo: r = 0.159, *p* = 0.033; CLEmo: r = 0.201, *p* = 0.007; EmoE: r = 0.196, *p* = 0.008), carbohydrates (DEmo: r = 0.234, *p* = 0.002; CLEmo: r = 0.271, *p* < 0.001; EmoE: r = 0.271, *p* < 0.001), fiber (DEmo: r = 0.158, *p* = 0.035; CLEmo: r = 0.186, *p* = 0.013; EmoE: r = 0.184, *p* = 0.013), and SFA (CLEmo: r = 0.163, *p* = 0.029; EmoE: r = 0.155, *p* = 0.038), as well as a lower intake of proteins (in percentage) (DEmo: r = −0.197, *p* = 0.008; CLEmo: r = −0.253, *p* = 0.001; EmoE: r = −0.246, *p* = 0.001). Interestingly, emotional eaters showed a significantly higher intake of grams of alcohol (CLEmo: r = 0.155, *p* = 0.037; EmoE: r = 0.154, *p* = 0.038). External eaters showed a significantly higher intake of energy (r = 0.209, *p* = 0.005), carbohydrates (r = 0.221, *p* = 0.003), total lipids (r = 0.147, *p* = 0.048), MUFA (r = 0.154, *p* = 0.039), SFA (r = 0.158, *p* = 0.034), and cholesterol (r = 0.202, *p* = 0.006), whereas restrained eaters showed a significantly lower intake of PUFA (r = −0.152, *p* = 0.042). In the women, we found no significant correlations between macronutrient intake and CLEmo or RE. Emotional eaters showed a significantly higher intake of energy (DEmo: r = 0.167, *p* = 0.010; EmoE: r = 0.138, *p* = 0.034), carbohydrates (DEmo: r = 0.161, *p* = 0.013; EmoE: r = 0.130, *p* = 0.047), fiber (DEmo: r = 0139, *p* = 0.033), total lipids (DEmo: r = 0.151, *p* = 0.020; EmoE: r = 0.129, *p* = 0.047), MUFA (DEmo: r = 0.161, *p* = 0.014; EmoE: r = 0.135, *p* = 0.039), SFA (DEmo: r = 0.147, *p* = 0.024; EmoE: r = 0.145, *p* = 0.026), and cholesterol only for DEmo (r = 0.133, *p* = 0.041). Women with external eating showed a significantly higher intake of energy (r = 0.190, *p* = 0.003), carbohydrates (r = 0.223, *p* = 0.001), fiber (r = 0.150, *p* = 0.022), total lipids (r = 0.143, *p* = 0.028), MUFA (r = 0.143, *p* = 0.028), and SFA (r = 0.145, *p* = 0.026), as well as a significantly lower intake of proteins (in percentage) (r = −0.132, *p* = 0.043).

**Figure 2:**
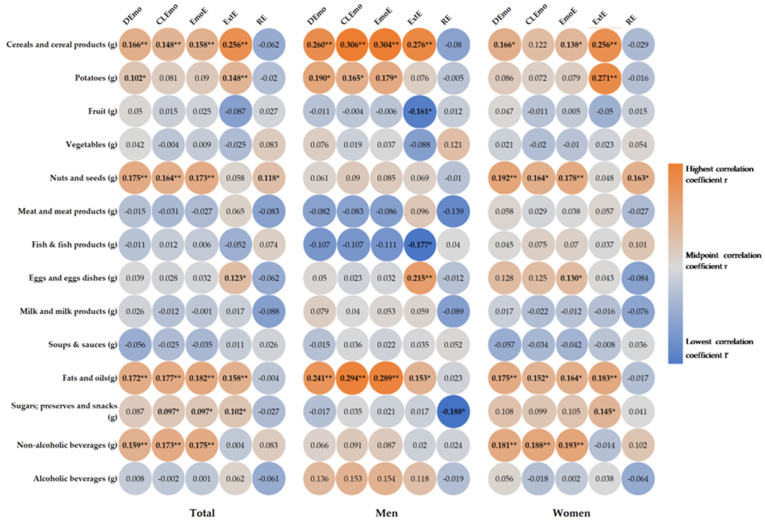
Heat map of correlation between daily food groups intake and eating behavior. Correlation coefficient r is presented in circle, and the strength of the correlation is depicted in color, with orange being the highest and blue the lowest correlation coefficient. * *p* < 0.05, ** *p* < 0.01. DEmo = diffuse emotions, CLEmo = clearly labelled emotions, EmoE = emotional eating, ExtE = external eating, RE = restrained eating.

Figure 2 shows the correlations between the intake of food groups and eating behavior. Significantly higher intakes of cereals (DEmo: r = 0.166, *p* = 0.001; CLEmo: r = 0.148, *p* = 0.003; EmoE: r = 0.158, *p* = 0.001), fats (DEmo: r = 0.172, *p* < 0.001; CLEmo: r = 0.177, *p* < 0.001; EmoE: r = 0.182, *p* < 0.001), nonalcoholic drinks (DEmo: r = 0.159, *p* = 0.001; CLEmo r = 0.173, *p* < 0.001; EmoE: r = 0.175, *p* < 0.001), nuts and seeds (DEmo: r = 0.175, *p* < 0.001; CLEmo: r = 0.164, *p* = 0.001; EmoE: r = 0.173, *p* < 0.001), potatoes (DEmo r = 0.102, *p* = 0.038), and sugars (CLEmo: r = 0.097, *p* = 0.049; EmoE: r = 097, *p* = 0.047) were recorded for emotional eaters. External eaters showed higher intakes of cereals (r = 0.256, *p* < 0.001), eggs (r = 0.123, *p* = 0.012), fats (r = 0.158, *p* = 0.001), potatoes (r = 0148, *p* = 0.002), and sugars (r = 0.102, *p* = 0.037). Restrained eaters also showed higher intakes of nuts and seeds (r = 0.118, *p* = 0.016). In men, emotional eaters showed higher intakes of cereals (DEmo: r = 0.260, *p* < 0.001; CLEmo: r = 0.306, *p* < 0.001; EmoE r = 0.304, *p* < 0.001), fats (DEmo: r = 0.241, *p* = 0.001; CLEmo: r = 0.294, *p* ≤ 0.001; EmoE: r = 0.289, *p* < 0.001), and potatoes (DEmo: r = 0.190 *p* = 0.010; CLEmo: r = 0.165, *p* = 0.027; EmoE: r = 0.179, *p* = 0.016). External eaters showed higher intakes of cereals (r = 0.276, *p* < 0.001), eggs (r = 0.215, *p* = 0.004), and fats (r = 0.153, *p* = 0.041), as well as a significantly lower intake of fish (r = −0.177, *p* = 0.018) and fruits (r = −0.161, *p* = 0.031). In the women, emotional eaters showed higher intakes of cereals (DEmo: r = 0.166, *p* = 0.011; EmoE: r = 0.138, *p* = 0.034), eggs (EmoE: r = 0.130, *p* < 0.001), fats (DEmo: r = 0.175, *p* = 0.007; CLEmo: r = 0.152, *p* = 0.020; EmoE: r = 0.164, *p* = 0.012), nonalcoholic drinks (DEmo: r = 0.181, *p* = 0.005; CLEmo: r = 0.188, *p* = 0.004; EmoE: r = 0.193, *p* = 0.003), nuts and seeds (DEmo: r = 0.192, *p* = 0.003; CLEmo: r = 0.164, *p* = 0.011; EmoE: r = 0.178, *p* = 0.006); external eaters showed higher intakes of cereals (r = 0.256, *p* < 0.001), fats (r = 0.183, *p* = 0.005), potatoes (r = 0.271, *p* < 0.001), and sugars (r = 0.145, *p* = 0.026). Restrained eaters showed significantly higher intakes of nuts and seeds (r = 0.163, *p* = 0.012).

## 4. Discussion

The main objective of this study was to investigate the relationship between eating behavior and dietary intake as well as the dietary habits of patients with T2DM. The results showed that individuals’ food intake was influenced by their eating behavior, with statistically significant correlations between food intake, and emotional and external eating behaviors. Particularly, our findings showed a high prevalence of individuals who were overweight and obesity, with over 90% of the population studied being overweight. Obesity is a major etiologic risk factor for T2DM and cardiometabolic disease [32], with overweight individuals accounting for up to 80% of diabetic patients [33]. Mihalache et al. found that more than half of the individuals living in a rural community in northeastern Romania were overweight or presented obesity [34]. This is in line with data from other countries showing an 86% prevalence of being overweight and obesity among individuals with T2DM in the UK, of which 52% had obesity [35].

In our study, patients had an average daily energy intake of 1584.78 kcal, similar to the results of other studies [36,37]. A recent systematic review showed that energy expenditure, measured by the doubly labeled water method, in individuals with T2DM averaged between 2159 and 3863 kcal, with no differences compared to the energy expenditure of individuals without diabetes [38]. Thus, the energy intake recorded in our study was lower than the average total energy expenditure. This may be explained by the fact that energy intake is underestimated when using instruments that collect retrospective dietary intake. Studies have confirmed the underestimation of energy intake in people with T2DM when similar methods of food intake assessment were used [36,39,40]. This underestimation can make it difficult to determine how nutrition and disease interact, a particularly important aspect when trying to identify the effect of nutrition on diabetes [41].

When assessing macronutrient intake, out study showed that the overall dietary intake was adequate, with a distribution of macronutrients relative to energy intake in accordance with existing guidelines [42,43,44]. According to currently available data, individual metabolic goals and preferences determine the optimal proportions of carbohydrate, protein, and fat in the diet [45]. The comparison between women and men in the study population showed that men have a higher intake of energy, proteins, total fat, saturated fatty acids, and cholesterol, and women consume more carbohydrates. This is consistent with previous work showing significant gender differences in dietary intake, particularly in the amounts of fat, and cholesterol intake [46]. For example, Leblanc et al. found that men had a higher energy intake, energy density, and percentage of energy from lipids and a lower percentage of energy from carbohydrates compared to women [47], results that are similar to those found in this study.

Our study participants achieved the recommended intake of fruits and vegetables, totaling more than 400 g daily, which is in line with WHO/FAO dietary recommendations for the prevention of chronic diseases [48]; however, the average consumption of fruits was higher than that of vegetables. Our study also showed significant sex differences between food groups with men having a higher intake of cereals, potatoes, meat, fish, eggs, soups and sauces, and alcoholic beverages compared to women. Vitale et al. also observed differences in food group intake between men and women with T2DM, with men consuming more starchy foods, soft drinks, and alcoholic beverages, and women consuming more plant-based foods (legumes, vegetables, and fruits), but also eggs, milk, vegetable oils, and added sugars [49]. These results highlight notable disparities in dietary preferences between men and women, emphasizing the need for gender-specific nutritional interventions.

In our study population, we found higher scores for external eating compared with emotional or restrained eating; thus, individuals tend to eat more in response to external stimuli (i.e., the taste of food), regardless of feelings of hunger or satiety [50]. Previous studies have associated higher scores for emotional, external, and restrained eating scales of the DEBQ questionnaire with unhealthy eating patterns, being overweight, and unfavorable emotional states [51,52,53]. Our research has also shown that women tend to score higher on emotional and restrained eating behaviors compared to men. Guerrero-Hreins et al. found that emotional eating scores differed for men and women, with a higher mean score for women compared to men [54]. Likewise, women, both those who were normal weight and overweight, had higher DEBQ scores than men for emotional eating and restrained eating, with no differences observed for external eating [51]. Although our study did not allow for an examination of changes in eating behavior as a function of age, previous work has shown differences between young and older adults when it comes to external eating [52]. Our results also showed higher scores for emotional and restrained eating in the urban environment. This can be explained by the influence of urbanization on eating habits. Urban economic status, food availability, and dietary transition to a highly palatable diet may lead to increased overeating, an increased weight and Body Mass Index, and subsequent body image concerns [55]. This finding may have important implications for the development of effective medical nutritional therapy interventions, particularly for women. It also highlights the need for further research in order to better understand the factors underlying gender differences and the role of urbanization in eating behavior.

The results of our study showed that there are significant differences in the associations between the type of eating (emotional, external, and restrained) and food intake. In particular, we found that emotional and external eating was correlated with a higher intake of energy, carbohydrates, fiber, and fats. The results are similar to other studies showing that emotional eating was associated with higher intakes of energy, fat, sugar, and alcohol, as well as fewer nutrients that are beneficial for health [15,56]. In a study by Laitinen et al., eating in response to negative emotions was positively associated with higher energy, fat, and sugar intakes in both men and women. Similarly, Torres and Nowson found that emotional eating was associated with a greater intake of energy-dense foods, particularly foods that are high in sugar and fat [57]. Stress is a negative factor that affects health, so eating food as a means of coping with stress and deriving pleasure, whether the food is sweet or not, provides comfort and attenuates the stress response [58]. Similar to our findings, another study also found that emotional eating is associated with a higher alcohol intake in men [59]. It is known that a high alcohol intake is a strong cardiovascular risk factor [60], thus it is important to assess emotional eating in patients with T2DM and to mitigate cardiovascular risk in these patients [54]. Several studies underscore the significant association between emotional eating and an increased consumption of energy-dense foods, leading to an increased risk of being overweight and obesity. In addition, the observed gender differences in emotional eating patterns highlight the importance of considering gender-specific factors when addressing eating behavior and its impact on weight and diabetes management.

Our study also found positive correlations, as well as differences, between the two genders in the relationship between external eating and food intake, with those with a higher external eating score having higher intakes of energy, carbohydrates, and fats. Previous studies have shown that external eating is associated with a higher intake of energy and energy-dense foods such as fat, sugar, and alcohol [61,62]. This is consistent with other studies that found a positive link between external eating and unhealthy food consumption [63], leading to weight gain and poor diabetes management. Collectively, these findings suggest that interventions targeting external eating behaviors may be effective in reducing energy intake and related health problems, and promoting weight loss, and healthier eating habits.

We also found that individuals with higher scores on the emotional eating scales had significantly higher intakes of cereals, fats, soft drinks, nuts and seeds, potatoes, and sugars. External eaters had a higher intake of cereals, eggs, fats, potatoes, and sugars, while those with restrained behaviors had a higher intake of nuts and seeds. In a study conducted by Burton et al., external factors were the main predictors of food cravings, and the response to external stimuli was mediated by cravings for specific food groups and was sex-dependent [64]. Likewise, our study showed that men reacted more easily to external stimuli compared to women, but differently for different food groups [64]. Restrained eating, as well as external eating, was associated with a low intake of healthy foods (fruits, vegetables, and unsweetened juices), while emotional eating predisposed individuals to an inadequate intake of fruits and vegetables [65]. In our study, we also found that women who exhibited emotional eating consumed significantly more non-alcoholic beverages. Sugar-sweetened beverages are among the nutritional factors that are most closely associated with cardiovascular risk, which is already high in T2DM patients [66]. The western dietary pattern, rich in animal products and carbonated beverages, has a negative impact on the anthropometric and metabolic parameters of T2DM patients [67]. Our study showed that individuals who scored higher on emotional or external eating considered food taste the major determinant of food choice, which is consistent with previous work [68,69]. Therefore, the development of nutritional strategies for patients with diabetes should take into account the fact that not incorporating taste into recommendations for nutritious foods (fruits, vegetables, and fish) could be a losing battle.

In our study, restrained eaters had a significantly lower intake of PUFA, which may suggest that restrained eating actually affects the intake of nutritious foods. Evidence suggests that restraint is a negative feature of eating behavior that leads to frustration, eating disorders, and ultimately weight gain [70,71]. Individuals with T2DM may perceive dietary recommendations as restrictive and not tailored to their individual needs. More restrained or rigid dietary recommendations, such as eliminating sugar, limiting foods containing carbohydrates, and reducing total fat in the diet, were more frequently described by individuals with diabetes as an important component of disease management compared with more flexible eating patterns [9]. The lack of other correlations with dietary intake in individuals with restrained behavior could suggest that some patients with diabetes may perceive that they are abstaining from food while, in reality, they are not.

### Strengths and Limitations

Our study demonstrates a direct relationship between dietary intake and eating behavior in individuals with T2DM and is one of the few studies evaluating these factors in a diabetic population. In addition, to our knowledge, this is the first study that uses the EPIC-FFQ and DEBQ questionnaires to assess the association between dietary intake and dietary behavior in patients with T2DM. There are scarce data in the literature on how eating behavior affects food intake in individuals with T2DM. Most studies that analyzed dietary intake in relation to at least one of the variables of eating behavior were conducted on children and adolescents [72,73,74,75,76,77]. Other studies have analyzed eating behavior in relation to food intake in bariatric patients [78,79], patients with metabolic syndrome [80], or the general population [65]. In the diabetic population, Van De Laar et al. studied energy and fat intakes and their association with eating behavior patterns [81]. Our study serves as an essential step in understanding the complex interplay between dietary intake and eating behavior and provides a baseline for future research or interventions.

Our study, however, has several limitations that warrant consideration. First, the nature of the cross-sectional study cannot capture changes over time and excludes the establishment of causal associations between the studied variables. Longitudinal studies tracking patients’ dietary intakes and eating behaviors over an extended period could provide more robust data and help analyze the dynamic relationship between variables as the disease progresses. Second, the methods used to collect the data, although validated, have multiple limitations. The FFQ can underestimate dietary intake and is subject to recall bias, as participants might inaccurately recall their dietary intake. Also, dietary behavior questionnaires, due to the sensitive subject matter that is covered, may cause respondents to omit or hide certain aspects out of fear of being judged. This could affect the validity and reliability of the results. In addition, our participants were recruited from a single healthcare center, limiting the generalizability of the findings to a broader population of T2DM patients. Lastly, our study did not control for various cofounding factors, such as physical activity, socioeconomic status, and comorbidities, which could impact both dietary intake and eating behavior. Adding objective measures, like biomarkers, or incorporating psychosocial factors such as stress and depression into the assessment could be helpful in validating the findings and better understanding their influence on dietary intake and eating behavior.

## 5. Conclusions

Our study showed a significant association between dietary intake and eating behavior in T2DM patients. Specifically, emotional and external eating were found to have a notable impact on dietary intake in the study population. On the other hand, restrained eating does not appear to influence food intake, suggesting that restraint is only a perceived attitude. This study’s findings suggest that dietary intake is affected by eating behavior and highlight the importance of considering emotional and external factors when studying individuals’ eating habits. By gaining deeper insight into eating behavior, we can better inform prevention efforts and clinical treatment plans to address the complex challenges related to diet in T2DM. Further research employing longitudinal designs and addressing potential biases and cofounding variables can further enhance our understanding of the complex interactions between dietary intake and eating behavior.

## Figures and Tables

**Table 1:** Descriptive characteristics of the population (*n* = 416).

	Total (*n* = 416)	Women (*n* = 236)	Men (*n* = 180)
Age (years)	62.64 ± 9.91	63.30 ± 9.31	62.11 ± 9.81
Weight (kg)	87.18 ± 17.59	81.12 ± 14.98 ^a^	95.08 ± 17.64 ^b^
BMI (kg/m^2^)	31.85 ± 5.33	31.98 ± 5.39	31.65 ± 5.25
Place of living			
Urban (%)	59.6 (*n* = 248)	58.1 (*n* = 137)	61.7 (*n* = 111)
Rural (%)	40.4 (*n* = 168)	41.9 (*n* = 99)	38.3 (*n* = 69)
Smoking status			
Non-smoking (%)	89.9 (*n* = 374)	95.3 (*n* = 225)	82.8 (*n* = 149)
Undeclared (%)	1.7 (*n* = 7)	1.7 (*n* = 4)	1.7 (*n* = 3)
BMI-status			
Normal weight (%)	7.5 (*n* = 31)	7.2 (*n* = 17)	7.8 (*n* = 14)
Overfweight (%)	33.3 (*n* = 138)	33.1 (*n* = 78)	33.9 (*n* = 61)
Obesity (%)	59.3 (*n* = 246)	59.7 (*n* = 141)	58.3 (*n* = 105)

Continuous variables are presented as mean ± SD, and categorical data as percentages (%) and numbers (*n*). SD = standard deviation; BMI = body mass index. ^a,b^ indicates significant difference between women and men (*p* < 0.05).

**Table 2:** Average daily intake of energy (kcal), macronutrients (grams and percent), and food groups in the studied population.

Daily Intake (Mean ± SD)	Total (*n* = 416)	Women (*n* = 236)	Men (*n* = 180)
Energy (kcal)	1584.78 ± 605.57	1509.01 ± 645.15 ^a^	1684.13 ± 535.05 ^b^
Carbohydrate (g)	195.08 ± 71.87	189.18 ± 75.43	202.80 ± 66.33
Carbohydrate (%)	50.05 ± 8.33	51.09 ± 8.23 ^a^	48.70 ± 8.28 ^b^
Fiber (g)	18.67 ± 6.1	18.52 ± 6.17	18.88 ± 6.01
Total lipids (g)	56.95 ± 31.36	54.25 ± 34.17 ^a^	60.50 ± 26.93 ^b^
Total lipids (%)	31.74 ± 6.81	31.23 ± 7.20	31.61 ± 6.06
MUFA (g)	20.38 ± 12.78	19.51 ± 14.21	21.53 ± 10.54
PUFA (g)	12.27 ± 6.81	11.81 ± 7.65	12.88 ± 5.48
SFA (g)	18.87 ± 10.82	17.79 ± 11.10 ^a^	20.28 ± 10.29 ^b^
Cholesterol (g)	322.47 ± 149.57	295.80 ± 140.30 ^a^	357.43 ± 154.45 ^b^
Proteins (g)	81.13 ± 28.44	76.73 ± 30.35 ^a^	86.90 ± 24.62 ^b^
Proteins (%)	20.77 ± 3.55	20.59 ± 3.54	21.03 ± 3.56
Cereals and cereal products	210.34 ± 111.04	198.50 ± 115.83 ^a^	225.86 ± 102.69 ^b^
Potatoes	63.18 ± 53.65	58.58 ± 38.26 ^a^	69.22 ± 68.46 ^b^
Fruit	336.51 ± 214.27	358.52 ± 224.76 ^a^	307.65 ± 196.60 ^b^
Vegetables	314.57 ± 135.92	316.08 ± 132.80	312.60 ± 140.25
Nuts and seeds	4.17 ± 9.10	5.04 ± 10.42 ^a^	3.03 ± 6.86 ^b^
Meat and meat products	123.42 ± 79.19	113.74 ± 84.00 ^a^	136.11 ± 70.62 ^b^
Fish & fish products	38.03 ± 35.87	37.48 ± 36.30 ^a^	38.74 ± 35.38 ^b^
Eggs and eggs dishes	21.16 ± 15.70	18.51 ± 11.02 ^a^	24.63 ± 19.77 ^b^
Milk and milk products	221.30 ± 180.55	210.69 ± 161.44	235.20 ± 202.53
Soups & sauces	224.16 ± 142.23	207.46 ± 91.05 ^a^	246.06 ± 187.53 ^b^
Fats and oils	7.76 ± 10.36	7.14 ± 7.92	8.58 ± 12.85
Sugars; preserves and snacks	16.98 ± 22.99	18.74 ± 25.53	14.69 ± 18.97
Non-alcoholic beverages	339.75 ± 237.76	362.71 ± 243.22 ^a^	309.65 ± 227.59 ^b^
Alcoholic beverages	32.08 ± 77.35	9.31 ± 25.22 ^a^	61.92 ± 107.04 ^b^

SD = standard deviation, MUFA = monounsaturated fatty acids, PUFA = polyunsaturated fatty acids, SFA = saturated fatty acids. The level of significance was considered at *p* < 0.05. ^a,b^ indicates significant difference between women and men.

**Table 3:** Mean scores for the five scales of DEBQ (*n* = 416).

	Score for DEmo	Score for CLEmo	Score for EmoE	Score for ExtE	Score for RE
Women	1.97 ± 1.07 **	1.93 ± 1.28 **	1.94 ± 1.17 **	2.44 ± 1.02	2.31 ± 0.97 **
Men	1.58 ± 0.77 **	1.49 ± 0.83 **	1.52 ± 0.78 **	2.36 ± 0.97	2.09 ± 0.90 **
BMI-status					
Normal weight	1.71 ± 0.94	1.42 ± 0.70	1.51 ± 0.71	2.40 ± 1.13	1.68 ± 0.62 **
Overweight	1.81 ± 0.96	1.75 ± 1.13	1.76 ± 1.04	2.43 ± 1.00	2.10 ± 0.85 **
Obesity	1.81 ± 0.98	1.78 ± 1.17	1.79 ± 1.08	2.39 ± 0.99	2.34 ± 1.00 **
Place of living					
Urban	1.90 ± 0.97 *	1.85 ± 1.12 *	1.87 ± 1.04 *	2.43 ± 0.98	2.29 ± 0.92 *
Rural	1.66 ± 0.95 *	1.57 ± 1.11 *	1.60 ± 1.03 *	2.36 ± 1.02	2.10 ± 0.97 *

DEBQ = Dutch Eating Behavior Questionnaire, DEmo = diffuse emotions, CLEmo = clearly labelled emotions, EmoE = emotional eating, ExtE = external Eating, RE = restrained Eating, BMI = Body Mass Index. Significantly different between groups: * *p* ≤ 0.05; ** *p* ≤ 0.01.

## Data Availability

Data is contained within the article.

## References

[1] Ortiz-Martínez M., González-González M., Martagón A.J., Hlavinka V., Willson R.C., Rito-Palomares M. (2022). Recent Developments in Biomarkers for Diagnosis and Screening of Type 2 Diabetes Mellitus. Curr. Diabetes Rep..

[2] Yun J.-S., Ko S.-H. (2021). Current Trends in Epidemiology of Cardiovascular Disease and Cardiovascular Risk Management in Type 2 Diabetes. Metabolism.

[3] Hallberg S.J., Gershuni V.M., Hazbun T.L., Athinarayanan S.J. (2019). Reversing Type 2 Diabetes: A Narrative Review of the Evidence. Nutrients.

[4] Magliano D., Boyko E.J. (2021). IDF Diabetes Atlas.

[5] Sun H., Saeedi P., Karuranga S., Pinkepank M., Ogurtsova K., Duncan B.B., Stein C., Basit A., Chan J.C.N., Mbanya J.C. (2022). IDF Diabetes Atlas: Global, Regional and Country-Level Diabetes Prevalence Estimates for 2021 and Projections for 2045. Diabetes Res. Clin. Pract..

[6] American Diabetes Association (2019). Standards of Medical Care in Diabetes—2019 Abridged for Primary Care Providers. Clin. Diabetes.

[7] Salvia M.G., Quatromoni P.A. (2023). Behavioral Approaches to Nutrition and Eating Patterns for Managing Type 2 Diabetes: A Review. Am. J. Med. Open.

[8] Stover P.J., Field M.S., Andermann M.L., Bailey R.L., Batterham R.L., Cauffman E., Frühbeck G., Iversen P.O., Starke-Reed P., Sternson S.M. (2023). Neurobiology of Eating Behavior, Nutrition and Health. J. Intern. Med..

[9] Yannakoulia M. (2006). Eating Behavior among Type 2 Diabetic Patients: A Poorly Recognized Aspect in a Poorly Controlled Disease. Rev. Diabet. Stud. RDS.

[10] Dakin C., Beaulieu K., Hopkins M., Gibbons C., Finlayson G., Stubbs R.J. (2023). Do Eating Behavior Traits Predict Energy Intake and Body Mass Index? A Systematic Review and Meta-Analysis. Obes. Rev..

[11] Cradock K.A., ÓLaighin G., Finucane F.M., McKay R., Quinlan L.R., Martin Ginis K.A., Gainforth H.L. (2017). Diet Behavior Change Techniques in Type 2 Diabetes: A Systematic Review and Meta-Analysis. Diabetes Care.

[12] Tak S.R., Hendrieckx C., Nefs G., Nyklíček I., Speight J., Pouwer F. (2015). The Association between Types of Eating Behaviour and Dispositional Mindfulness in Adults with Diabetes. Results from Diabetes MILES. The Netherlands. Appetite.

[13] Park M., Quinn L., Park C., Martyn-Nemeth P. (2018). Pathways of the Relationships among Eating Behavior, Stress, and Coping in Adults with Type 2 Diabetes: A Cross-Sectional Study. Appetite.

[14] Koopman A.D.M., Vd Ven M., Beulens J.W., Welschen L.M., Elders P.J., Nijpels G., Rutters F. (2018). The Association between Eating Traits and Weight Change after a Lifestyle Intervention in People with Type 2 Diabetes Mellitus. J. Diabetes Res..

[15] Betancourt-Núñez A., Torres-Castillo N., Martínez-López E., De Loera-Rodríguez C.O., Durán-Barajas E., Márquez-Sandoval F., Bernal-Orozco M.F., Garaulet M., Vizmanos B. (2022). Emotional Eating and Dietary Patterns: Reflecting Food Choices in People with and without Abdominal Obesity. Nutrients.

[16] Van Strien T., Van De Laar F.A. (2008). Intake of Energy Is Best Predicted by Overeating Tendency and Consumption of Fat Is Best Predicted by Dietary Restraint: A 4-Year Follow-up of Patients with Newly Diagnosed Type 2 Diabetes. Appetite.

[17] Huisman S.D., Hendrieckx C., Bot M., Pouwer F., Nefs G. (2023). Prevalence, Associations and Health Outcomes of Binge Eating in Adults with Type 1 or Type 2 Diabetes: Results from Diabetes MILES–The Netherlands. Diabet. Med..

[18] Forouhi N.G., Misra A., Mohan V., Taylor R., Yancy W. (2018). Dietary and Nutritional Approaches for Prevention and Management of Type 2 Diabetes. Br. Med. J..

[19] Al-Salmi N., Cook P., D’Souza M.S. (2022). Diet Adherence among Adults with Type 2 Diabetes Mellitus: A Concept Analysis. Oman Med. J..

[20] American Diabetes Association (2021). Prevention or Delay of Type 2 Diabetes: Standards of Medical Care in Diabetes—2021. Diabetes Care.

[21] Ajala O., English P., Pinkney J. (2013). Systematic Review and Meta-Analysis of Different Dietary Approaches to the Management of Type 2 Diabetes. Am. J. Clin. Nutr..

[22] Esposito K., Maiorino M.I., Bellastella G., Chiodini P., Panagiotakos D., Giugliano D. (2015). A Journey into a Mediterranean Diet and Type 2 Diabetes: A Systematic Review with Meta-Analyses. BMJ Open.

[23] Schwingshackl L., Hoffmann G. (2014). Mediterranean Dietary Pattern, Inflammation and Endothelial Function: A Systematic Review and Meta-Analysis of Intervention Trials. Nutr. Metab. Cardiovasc. Dis..

[24] Inagaki S., Matsuda T., Muramae N., Abe K., Kato K. (2022). Diabetes-Related Shame among People with Type 2 Diabetes: An Internet-Based Cross-Sectional Study. BMJ Open Diabetes Res. Care.

[25] de Queiroz F.L.N., Raposo A., Han H., Nader M., Ariza-Montes A., Zandonadi R.P. (2022). Eating Competence, Food Consumption and Health Outcomes: An Overview. Int. J. Environ. Res. Public Health.

[26] Przybyłowicz K.E., Danielewicz A. (2022). Eating Habits and Disease Risk Factors. Nutrients.

[27] World Health Organization, WHO Expert Committee on Physical Status (1995). Physical Status: The Use and Interpretation of Anthropometry: Report of a WHO Expert Committee.

[28] Mulligan A.A., Luben R.N., Bhaniani A., Parry-Smith D.J., O’Connor L., Khawaja A.P., Forouhi N.G., Khaw K.-T. (2014). A New Tool for Converting Food Frequency Questionnaire Data into Nutrient and Food Group Values: FETA Research Methods and Availability. BMJ Open.

[29] Gherasim A., Arhire L.I., Niță O., Strateanu R., Oprescu A.C., Graur M., Mihalache L. (2015). Can the Epic Food Frequency Questionnaire Be Applied to the Population in Romania. Rev. Med. Chir. Soc. Med. Nat. Iasi.

[30] van Strien T., Frijters J.E., Bergers G.P., Defares P.B. (1986). The Dutch Eating Behavior Questionnaire (DEBQ) for Assessment of Restrained, Emotional, and External Eating Behavior. Int. J. Eat. Disord..

[31] Arhire L.I., Niță O., Popa A.D., Gal A.-M., Dumitrașcu O., Gherasim A., Mihalache L., Graur M. (2021). Validation of the Dutch Eating Behavior Questionnaire in a Romanian Adult Population. Nutrients.

[32] Mihalache L., Graur L.I., Popescu D.S., Niţă O., Graur M. (2012). Anthropometric Parameters—Predictive Factors for Cardio-Metabolic Diseases. Rev. Med. Chir. Soc. Med. Nat. Iasi.

[33] World Health Organization (2022). Fact Sheet about Diabetes.

[34] Mihalache L. (2012). The Prevalence of the Metabolic Syndrome and Its Components in a Rural Community. Acta Endocrinol. Buchar..

[35] Daousi C., Casson I.F., Gill G.V., MacFarlane I.A., Wilding J.P.H., Pinkney J.H. (2006). Prevalence of Obesity in Type 2 Diabetes in Secondary Care: Association with Cardiovascular Risk Factors. Postgrad. Med. J..

[36] Nascimento A.G., Grassi T., Reischak de Oliveira A., Steemburgo T. (2021). Under-reporting of the Energy Intake in Patients with Type 2 Diabetes. J. Hum. Nutr. Diet..

[37] Thewjitcharoen Y., Chotwanvirat P., Jantawan A., Siwasaranond N., Saetung S., Nimitphong H., Himathongkam T., Reutrakul S. (2018). Evaluation of Dietary Intakes and Nutritional Knowledge in Thai Patients with Type 2 Diabetes Mellitus. J. Diabetes Res..

[38] Katsukawa F. (2021). Energy Requirements for Older Patients with Type 2 Diabetes: A Narrative Review of the Current Findings and Future Tasks. Nutrients.

[39] McKenzie B.L., Coyle D.H., Burrows T., Rosewarne E., Peters S.A.E., Carcel C., Collins C.E., Norton R., Woodward M., Jaacks L.M. (2020). Gender Differences in the Accuracy of Dietary Assessment Methods to Measure Energy Intake in Adults: Protocol for a Systematic Review and Meta-Analysis. BMJ Open.

[40] Goode J.P., Smith K.J., Kilpatrick M., Breslin M., Oddy W.H., Dwyer T., Venn A.J., Magnussen C.G. (2021). Retrospectively Estimating Energy Intake and Misreporting from a Qualitative Food Frequency Questionnaire: An Example Using Australian Cohort and National Survey Data. Front. Nutr..

[41] Miyazawa I., Morino K., Fuse K., Kondo K., Ohi A., Nishida K., Kurihara M., Yasuhara S., Nakanishi N., Nishida Y. (2020). Impact of Obesity on Underreporting of Energy Intake in Type 2 Diabetic Patients: Clinical Evaluation of Energy Requirements in Patients with Diabetes Mellitus (CLEVER-DM) Study. Clin. Nutr. ESPEN.

[42] Davies M.J., D’Alessio D.A., Fradkin J., Kernan W.N., Mathieu C., Mingrone G., Rossing P., Tsapas A., Wexler D.J., Buse J.B. (2018). Management of Hyperglycemia in Type 2 Diabetes, 2018. A Consensus Report by the American Diabetes Association (ADA) and the European Association for the Study of Diabetes (EASD). Diabetes Care.

[43] Evert A.B., Dennison M., Gardner C.D., Garvey W.T., Lau K.H.K., MacLeod J., Mitri J., Pereira R.F., Rawlings K., Robinson S. (2019). Nutrition Therapy for Adults with Diabetes or Prediabetes: A Consensus Report. Diabetes Care.

[44] American Diabetes Association (2021). Classification and Diagnosis of Diabetes: Standards of Medical Care in Diabetes—2021. Diabetes Care.

[45] Gray A., Threlkeld R.J., Feingold K.R., Anawalt B., Blackman M.R., Boyce A., Chrousos G., Corpas E., de Herder W.W., Dhatariya K., Dungan K., Hofland J. (2000). Nutritional Recommendations for Individuals with Diabetes. Endotext.

[46] Abassi M.M., Sassi S., El Ati J., Ben Gharbia H., Delpeuch F., Traissac P. (2019). Gender Inequalities in Diet Quality and Their Socioeconomic Patterning in a Nutrition Transition Context in the Middle East and North Africa: A Cross-Sectional Study in Tunisia. Nutr. J..

[47] Leblanc V., Bégin C., Corneau L., Dodin S., Lemieux S. (2015). Gender Differences in Dietary Intakes: What Is the Contribution of Motivational Variables?. J. Hum. Nutr. Diet..

[48] Nishida C., Uauy R., Kumanyika S., Shetty P. (2004). The Joint WHO/FAO Expert Consultation on Diet, Nutrition and the Prevention of Chronic Diseases: Process, Product and Policy Implications. Public Health Nutr..

[49] Vitale M., Masulli M., Cocozza S., Anichini R., Babini A.C., Boemi M., Bonora E., Buzzetti R., Carpinteri R., Caselli C. (2016). Sex Differences in Food Choices, Adherence to Dietary Recommendations and Plasma Lipid Profile in Type 2 Diabetes—The TOSCA.IT Study. Nutr. Metab. Cardiovasc. Dis..

[50] Herman C.P., Polivy J. (2008). External Cues in the Control of Food Intake in Humans: The Sensory-Normative Distinction. Physiol. Behav..

[51] Andrés A., Oda-Montecinos C., Saldaña C. (2017). Eating Behaviors in a Male and Female Community Sample: Psychometric Properties of the DEBQ. Ter. Psicológica.

[52] Daly A., O’Sullivan E., McNulty B., Walton J., Kearney J. (2020). Age, Sex & BMI Are Associated with Different Eating Behaviour Styles in Irish Teens. Proc. Nutr. Soc..

[53] Mason T.B., Lewis R.J. (2014). Profiles of Binge Eating: The Interaction of Depressive Symptoms, Eating Styles, and Body Mass Index. Eat. Disord..

[54] Guerrero-Hreins E., Stammers L., Wong L., Brown R.M., Sumithran P. (2022). A Comparison of Emotional Triggers for Eating in Men and Women with Obesity. Nutrients.

[55] Gorrell S., Trainor C., Le Grange D. (2019). The Impact of Urbanization on Risk for Eating Disorders. Curr. Opin. Psychiatry.

[56] Aguiar-Bloemer A.C., Diez-Garcia R.W. (2018). Influence of Emotions Evoked by Life Events on Food Choice. Eat. Weight Disord. Stud. Anorex. Bulim. Obes..

[57] Torres S.J., Nowson C.A. (2007). Relationship between Stress, Eating Behavior, and Obesity. Nutrition.

[58] Standen E.C., Finch L.E., Tiongco-Hofschneider L., Schopp E., Lee K.M., Parker J.E., Bamishigbin O.N., Tomiyama A.J. (2022). Healthy versus Unhealthy Comfort Eating for Psychophysiological Stress Recovery in Low-Income Black and Latinx Adults. Appetite.

[59] Laitinen J., Ek E., Sovio U. (2002). Stress-Related Eating and Drinking Behavior and Body Mass Index and Predictors of This Behavior. Prev. Med..

[60] Piano M.R. (2017). Alcohol’s Effects on the Cardiovascular System. Alcohol Res. Curr. Rev..

[61] Anschutz D.J., Van Strien T., Van De Ven M.O.M., Engels R.C.M.E. (2009). Eating Styles and Energy Intake in Young Women. Appetite.

[62] Paans N.P.G., Gibson-Smith D., Bot M., van Strien T., Brouwer I.A., Visser M., Penninx B.W.J.H. (2019). Depression and Eating Styles Are Independently Associated with Dietary Intake. Appetite.

[63] Shukri M., Jones F., Conner M. (2018). Relationship between Work-Family Conflict and Unhealthy Eating: Does Eating Style Matter?. Appetite.

[64] Burton P., Smit H.J., Lightowler H.J. (2007). The Influence of Restrained and External Eating Patterns on Overeating. Appetite.

[65] Małachowska A., Jeżewska-Zychowicz M., Gębski J. (2021). Polish Adaptation of the Dutch Eating Behaviour Questionnaire (DEBQ): The Role of Eating Style in Explaining Food Intake—A Cross-Sectional Study. Nutrients.

[66] Narain A., Kwok C.S., Mamas M.A. (2016). Soft Drinks and Sweetened Beverages and the Risk of Cardiovascular Disease and Mortality: A Systematic Review and Meta-Analysis. Int. J. Clin. Pract..

[67] Gherasim A., Arhire L.I., Nita O., Oprescu A.C., Gavril R.S., Mitu O., Graur M., Mihalache L. (2022). Dietary Patterns in Romanian Patients with Type 2 Diabetes Mellitus. Med.-Surg. J..

[68] Liem D.G., Russell C.G. (2019). The Influence of Taste Liking on the Consumption of Nutrient Rich and Nutrient Poor Foods. Front. Nutr..

[69] Kabir A., Miah S., Islam A. (2018). Factors Influencing Eating Behavior and Dietary Intake among Resident Students in a Public University in Bangladesh: A Qualitative Study. PLoS ONE.

[70] Lowe M.R., Doshi S.D., Katterman S.N., Feig E.H. (2013). Dieting and Restrained Eating as Prospective Predictors of Weight Gain. Front. Psychol..

[71] Arhire L.I. (2015). Orthorexia Nervosa: The Unhealthy Obsession for Healthy Food. Med.-Surg. J..

[72] Quattlebaum M., Wilson D.K., Sweeney A.M., Zarrett N. (2021). Moderating Effects of Parental Feeding Practices and Emotional Eating on Dietary Intake among Overweight African American Adolescents. Nutrients.

[73] Bell B.M., Spruijt-Metz D., Naya C.H., Lane C.J., Wen C.K.F., Davis J.N., Weigensberg M.J. (2021). The Mediating Role of Emotional Eating in the Relationship between Perceived Stress and Dietary Intake Quality in Hispanic/Latino Adolescents. Eat. Behav..

[74] Plaza-Diaz J., Flores-Rojas K., de la Torre-Aguilar M.J., Gomez-Fernández A.R., Martín-Borreguero P., Perez-Navero J.L., Gil A., Gil-Campos M. (2021). Dietary Patterns, Eating Behavior, and Nutrient Intakes of Spanish Preschool Children with Autism Spectrum Disorders. Nutrients.

[75] Schneider-Worthington C.R., Smith K.E., Roemmich J.N., Salvy S.-J. (2022). External Food Cue Responsiveness and Emotional Eating in Adolescents: A Multimethod Study. Appetite.

[76] Dubois L., Bédard B., Goulet D., Prud’homme D., Tremblay R.E., Boivin M. (2022). Eating Behaviors, Dietary Patterns and Weight Status in Emerging Adulthood and Longitudinal Associations with Eating Behaviors in Early Childhood. Int. J. Behav. Nutr. Phys. Act..

[77] Maneschy I., Moreno L.A., Ruperez A.I., Jimeno A., Miguel-Berges M.L., Widhalm K., Kafatos A., Molina-Hidalgo C., Molnar D., Gottrand F. (2022). Eating Behavior Associated with Food Intake in European Adolescents Participating in the HELENA Study. Nutrients.

[78] Al-Najim W., Docherty N.G., le Roux C.W. (2018). Food Intake and Eating Behavior After Bariatric Surgery. Physiol. Rev..

[79] Sarwer D.B., Dilks R.J., West-Smith L. (2011). Dietary Intake and Eating Behavior after Bariatric Surgery: Threats to Weight Loss Maintenance and Strategies for Success. Surg. Obes. Relat. Dis..

[80] Morita A., Aiba N., Miyachi M., Watanabe S., Saku Cohort Study Group (2020). The Associations of Eating Behavior and Dietary Intake with Metabolic Syndrome in Japanese: Saku Cohort Baseline Study. J. Physiol. Anthropol..

[81] Van De Laar F.A., Van De Lisdonk E.H., Lucassen P.L.B.J., Stafleu A., Mulder J., Van Den Hoogen H.J.M., Rutten G.E.H.M., Van Weel C. (2006). Eating Behaviour and Adherence to Diet in Patients with Type 2 Diabetes Mellitus. Diabet. Med..

